# Huperzine A associated with improved early postoperative cognitive function after neurosurgery under general anesthesia: a randomized clinical trial

**DOI:** 10.1097/JS9.0000000000004478

**Published:** 2025-12-09

**Authors:** Zhongding Zhang, Yiman Shen, Juan Li, Yinda Tang, Ping Zhou, Tingting Ying, Ruoping Mo, Ziang Ru, Guanjia Zhao, Jin Zhu, Shiting Li, Hua Zhao

**Affiliations:** a Department of Neurosurgery, Xinhua Hospital Affiliated to Shanghai Jiao Tong University School of Medicine, Shanghai, China; b Academician & Expert Workstations, Wanbangde pharmaceutical Group Co., Ltd., Wenling, Zhejiang Province, China

**Keywords:** acetylcholinesterase inhibitor, huperzine A, neurosurgery, postoperative neurocognitive disorders, general anesthesia

## Abstract

**Importance:**

Postoperative neurocognitive disorders (PNCD) are a group of complications following surgery and anesthesia. Huperzine A, an acetylcholine esterase (AChE) inhibitor used to treat cognitive disorders, is promising in improving the postoperative cognitive function.

**Objective:**

To assess the efficacy of huperzine A injection on improving early postoperative cognitive function in patients undergoing neurosurgical procedures under general anesthesia.

**Design, setting, and participants:**

This parallel group, randomized trial was conducted at a neurosurgical department of a tertiary hospital in China from May, 2021 to February, 2023. Adult patients scheduled for selective neurosurgical operations requiring general anesthesia were recruited. Participants were randomized 1:1 to postoperative huperzine A injections or standard care without additional pharmacological intervention.

**Intervention:**

In the intervention group, 0.2 mg huperzine A was intramuscularly injected after tracheal extubation, and at 10:00 ± 2 h on the first, second, and third postoperative days, respectively. Patients in the nonintervention group did not receive huperzine A injection after surgery.

**Main outcomes and measures:**

The evaluation was performed at 6 h ± 2 h, 24 h ± 2 h, 48 h ± 2 h, 72 h ± 2 h, 96 h ± 2 h after the first administration. The primary outcome was the area under the curve in 0 to 96 hours postoperatively (AUC_0–96h_) of the mini-mental state examination (MMSE; score range: 0–30, with the higher score indicating better cognitive function) in total score and score changes from baseline, which represented total “cognitive burden” over the critical first 96 postoperative hours.

**Results:**

The trial recruited 127 patients, of which 123 patients were included in the full analysis set (76 females [61.8%]; mean [SD] age, 59.6 [12.6] years). There were 58 patients in intervention group (40 females [69.0%]; mean [SD] age, 59.6 [11.9] years) and 65 in nonintervention group (36 females [55.4%]; mean [SD] age, 59.7 [13.2] years). Preoperative MMSE baseline were comparable between the two groups (intervention group: 28.5 [2.1]; nonintervention group: 28.0 [2.7], *P* = 0.608). Postoperatively, the intervention group demonstrated a significantly less decline in cognitive function, as measured by the mean (SD) AUC_0–96h_ of the MMSE total scores (2770.67 [150.67]) compared with nonintervention group (2651.34 [257.41]) (*P* = 0.010). Accordingly, the mean MMSE change from baseline in the AUC_0-96h_ was also significantly better in the intervention group (−2.67 [170.29]) than in the non-intervention group (−53.05 [164.04]) (P = 0.021).

**Conclusions and relevance:**

In adults undergoing neurosurgery with general anesthesia, postoperative administration of huperzine A associated with significantly improved MMSE scores over the 96-hour postoperative period.

## Introduction

Postoperative neurocognitive disorder (PNCD) are a group of common complications following surgery and anesthesia, characterized by impairments in memory, attention, language comprehension, and social skills, which can lead to prolonged hospital stays, reduced life quality, and even increased morbidity^[1–3]^. With an estimated incidence of 10-65%, PNCD affects patients of all ages and leads to prolonged hospital stays, reduced quality of life, and increased morbidity, thereby posing a significant public health and economic burden^[4–8]^. As the global population ages and surgical volumes in older adults rise, the prevalence and impact of PNCD are expected to increase, making its management a critical focus in perioperative care.

Anesthesiologists and pharmacologists are actively seeking effective pharmacological interventions to mitigate PNCD. Drugs, such as Esketamine, Dexmedetomidine, and Haloperidol have been investigated in clinical trials, but did not demonstrate obvious benefits^[9–12]^. Huperzine A is a natural, highly effective, and reversible selective acetylcholinesterase inhibitors (AchEIs) derived from the plant Huperzia serrata, which belongs to the family Huperziaceae^[13,14]^. It primarily functions by inhibiting the activity of acetylcholinesterase (AChE), reducing the breakdown of acetylcholine (Ach) at the postsynaptic membrane, and activating corresponding receptors[15]. Animal experiments and clinical studies have confirmed that huperzine A can easily pass through the blood–brain barrier (BBB), with the ability of enhancing memory retention and recall, as well as reversing or alleviating cognitive deficits, which exhibits minimal peripheral cholinergic side effects compared to other AChEIs[16]. Besides, huperzine A can inhibit inflammatory response and protect neurons by decreasing the expression of IL-1β, TNF-α protein and inhibiting transcriptional activation of NF-κB signaling[17]. In 1997, the U.S. FDA approved it as a dietary supplement for improving memory decline. Huperzine A’s role in improving cognition and treating neurodegenerative diseases such as Alzheimer’s disease has been widely reported without significant side effects^[18–20]^. We hypothesized that postoperative administration of Huperzine A would reduce early cognitive decline in patients undergoing neurosurgery under general anesthesia, and therefore conducted this study.

This study is a single-center, prospective, open, and randomized real-world study aimed at observing whether huperzine A injection can reduce cognitive function impairment and prevent the occurrence of PNCD in neurosurgical patients. Here, we report the final results of clinical efficacy and safety of huperzine A in preventing PNCD. The study is compliant with the TITAN Guidelines 2025[21].

## Methods

### Trial design

This parallel-group randomized controlled trial was conducted in Xinhua Hospital Affiliated to Shanghai Jiao Tong University School of Medicine from May, 2021 to February, 2023. The trial was approved by the Ethics Committee of the Xinhua Hospital Affiliated to Shanghai Jiao Tong University School of Medicine and was registered before patient enrollment at Chinese Clinical Trial Registry (Registration number: [ChiCTR2000034272]). The protocol translated from Chinese is available in Supplemental Digital Content S1-protocal available at: http://links.lww.com/JS9/G253. Written informed consent was obtained from all subjects. The study was performed in accordance with the principles of the Declaration of Helsinki and was in line with the Consolidated Standards of Reporting Trials (CONSORT) reporting guideline^[22,23]^.

**Highlights**

- Postoperative neurocognitive disorders remain a significant clinical challenge, particularly in neurosurgical populations exposed to prolonged anesthesia and surgical stress. However, there is still no effective drug to treat postoperative neurocognitive disorders.
- Our randomized clinical trial demonstrates that huperzine A mitigate early postoperative cognitive impairment, potentially via acetylcholinesterase inhibition, with a favorable safety profile in neurosurgical patients.

### Participants and selection criteria

A total of 127 patients who fulfilled the inclusion criteria were recruited from a cohort of 191 patients screened at Xinhua Hospital Affiliated to Shanghai Jiao Tong University School of Medicine. The inclusion criteria were (1) meet the indications for neurosurgery under general anesthesia; (2) agrees to participate in this study, and either the patient or their legally authorized representative signs the informed consent form before the trial.

Exclusion criteria were (1) unable to tolerate the trial or cooperate with the examination for various reasons, including aphasia, sensory and auditory dysfunction, etc.; (2) known allergy to huperzine A; (3) history of mental illness, long-term use of sedatives, antidepressants, or similar medications; (4) history of alcohol or substance abuse; (5) severe systemic diseases, particularly cardiovascular diseases, including myocardial infarction, heart failure, unstable angina, or a history of bradycardia; (6) history of mechanical intestinal obstruction, epilepsy, renal insufficiency, bronchial asthma, or similar conditions; and (7) positive result in infectious disease screening.

### Randomization and treatment

Patients were randomly assigned to huperzine A injection group (intervention group) or nonintervention group in a 1:1 ratio. SAS software (version 9.4) was used for randomization with the disease types (intracranial space-occupying lesion such as tumors vs functional diseases) defined as stratification factors. A random allocation table is generated, ensuring that the results of the randomization are reproducible. Randomization parameters and seed numbers are stored in the random allocation table.

Participants in the intervention group were administered 0.2 mg of huperzine A via injection immediately after extubation, and again at 10:00 AM (± 2 hours) on the first, second, and third days postsurgery. The nonintervention group received standard postoperative care without additional interventions.

### Outcomes measures

The patients underwent cognitive status assessment before and after surgery by independent evaluators who do not know the grouping. The primary outcome was the cognitive status of the patient within 96 hours after surgery using the MMSE score[24]. The Mandarin version of MMSE, which contained items of time orientation, place orientation, verbal immediate memory, attention and calculation, recall (short-term memory), object naming, verbal repetition, reading comprehension, lalognosis, verbal expression, and graphic drawing, were collected at the baseline, at 6 hours, 24 hours, 48 hours, 72 hours, and 96 hours after surgery by the trained investors (± 2 h). The area under the curve from 0 to 96 hours (AUC_0–96h_) of MMSE were used to compare between two groups. The secondary outcomes included Montreal Cognitive Assessment Scale (MoCA) score, incidence of PNCD (postoperative MMSE total score < 27 at any visit), postoperative hospital stay, and the incidence of adverse events during treatment. We chose the MMSE as the primary outcome measure due to its simplicity and widespread use in China. Its ease of administration makes it particularly suitable for rapid assessment in busy clinical settings. In our trial, MMSE assessments were prioritized over the MoCA. For all participants, MMSE assessments were discontinued once a score of 30 was achieved after the first administration, and missing timepoints postachievement of 30 were imputed as 30. Additionally, for participants under 65 years old, if the MMSE score after administration was greater than or equal to the screening period score, subsequent MMSE evaluations were terminated, with missing efficacy data imputed using the screening period score. MoCA assessments and missing data imputation procedures are detailed in the protocol and Supplemental Digital Content eMethod available at: http://links.lww.com/JS9/G254 in supplement files.

Exploratory indicators included the plasma concentration of huperzine A, acetylcholinesterase activity in blood cells, and serum levels of NSE (neuron-specific enolase), S100B (S100 calcium-binding protein B), IL-6 (interleukin 6), and TNF-α (tumor necrosis factor alpha). Venous blood was collected and treated with EDTA for anticoagulation. Subsequently, plasma and blood cells were separated. Additional separate separations were conducted for blood and serum. huperzine A levels were measured in plasma. Acetylcholinesterase activity was assessed in blood cells. NSE, S100B, IL-6, and TNF-α levels were quantified in serum. Distribution of blood collection time points were detailed in protocol.

### Sample size calculation

The sample size was calculated for a two-sample, one-sided *t*-test with a significance level (α) of 0.025. Based on prior studies, we conservatively estimated a 0.7 mean MMSE score change in the intervention group and a 1.2 change mean MMSE score change in the nonintervention group, yielding an effect size of 0.5 (difference in means)[25]. A common standard deviation of 1.7 was assumed for both groups. To achieve 90% statistical power (1−β = 0.9), ensuring a high probability of detecting the specified effect if it exists, 199 participants per group were required. Accounting for an estimated 20% attrition rate, the final target enrollment was set at 240 participants per group (total *N* = 480).

### Termination of trial

The trial was terminated prematurely in February, 2023 due to a confluence of factors: COVID-19 pandemic-related disruptions and the prespecified efficacy criteria being met at interim analysis. The researcher, sponsor and the data and safety monitoring board jointly passed this resolution.

### Statistical analysis

All statistical analyses were performed using SAS 9.4 software (SAS Institute Inc.) and R software, version 4.3.3 (R Core Development Team). Quantitative data were compared between groups by *t*-test or nonparametric test, depending on normality. Qualitative data were using chi-square test or Fisher exact calculation, as appropriate. The area under the curve from 0 to 96 hours (AUC_0–96h_) of score were used to compare MMSE and MoCA outcomes between two groups. Generalized Linear models were used to estimate difference of MMSE change and 95% CIs for the association between age, gender, BMI, and operation procedure. The subgroup analysis of PNCD based on MMSE scores was performed using logistic regression model. The correlation analysis of exploratory indicators was carried out. A *P-*value lower than 0.05 (2-tailed) was deemed significant.

The adverse events analysis was analyzed according to the actual group. Due to the influence of the COVID-19 epidemic and the political and legal containment measures during the trial, part of the MMSE and MoCA scores evaluation could not be performed, and the missing values were first imputed according to the Protocol (Supplemental Digital Content S1-protocal available at: http://links.lww.com/JS9/G253). The missing data violating the standard of imputation were excluded in main analysis. In the sensitivity analyses, missing values were imputed using the last observation carried forward method (Supplemental Digital Content eMethod available at: http://links.lww.com/JS9/G254 and Supplemental Digital Content eTable 1 available at: http://links.lww.com/JS9/G247 in supplement files). Following the completion of the trial, a post-hoc power analysis of main results was conducted based on the observed effect sizes and sample size at a two-sided alpha level of 0.05.

## Results

### Patients

A total of 191 patients were screened, of which 127 participants were assessed eligible and randomized to intervention group and nonintervention group. Excluding the four patients in the intervention group withdrawn before the treatment and receive no doses of the injection, 123 patients were included in the full analysis set (58 in intervention group and 65 in nonintervention group). The participants included 47 males (38.2%) and 76 females (61.8%), and all of them were Asian patients. The mean age (SD) of the subjects was 59.63 (12.57) years. The mean height (SD) was 1.64 (0.08) m, and the mean weight (SD) was 65.14 (11.23) kg.

**Figure 1.**
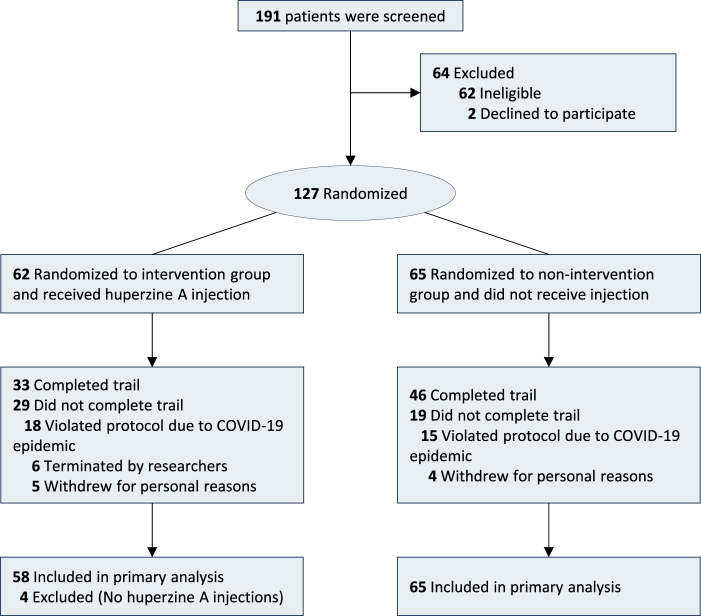
Trial flow diagram.

Excluding patients who withdrew before treatment initiation, all patients in the intervention group received at least one dose of medication. Compliance rates across four administrations were: 100% (58/58) on the day of surgery, 96.6% (56/58) of second dose, 89.7% (52/58) of the third dose, and 86.2% (50/58) of the fourth dose. One participant in the nonintervention group at randomization was mistakenly administered to the intervention group in the trial, and the efficacy analysis was still performed according to the planned group according to the Intention to Treat (ITT) principle. The baseline data between the two groups is comparable (Table 1). The study flowchart is presented in Figure 1.

**Table 1:** Baseline demographic characteristics of the patients^a^.

Characteristics	Intervention group (*n* = 58)	Nonintervention group (*n* = 65)
Sex		
Female	40 (69.0)	36 (55.4)
Male	18 (31.0)	29 (44.6)
Age, years	59.6 (52.0 to 67.0)	59.7 (52.0 to 69.0)
Height, m	1.64 (1.58 to 1.70)	1.64 (1.58 to 1.70)
Weight, kg	64.0 (53.0 to 75.0)	66.1 (59.0 to 71.0)
BMI, kg/m^2^	23.8 (21.3 to 26.1)	24.4 (22.2 to 26.6)
Neurosurgical procedure^b^		
MVD	46 (79.3)	52 (80.0)
Lesions resection	12 (20.7)	13 (20.0)
Operation time	126.2 (95.0 to 141.3)	130.5 (95.0 to 145.0)
Anesthesia time	156.2 (120.0 to 172.0)	153.6 (122.5 to 170.0)

MVD: microvascular decompression

^a^ Values are presented as No. (%) of patients or interquartile range (IQR) in parenthesis.

^b^ Microvascular decompression surgery with retrosigmoid craniotomy are all minimally invasive functional surgeries to treat benign diseases including trigeminal neuralgia, hemifacial spasm and glossopharyngeal neuralgia. Lesions resection surgeries were for intracranial space-occupying lesion (such as resection of tumor, arachnoid cyst, cavernous hemangioma and etc.).

### Primary outcome

**Figure 2.**
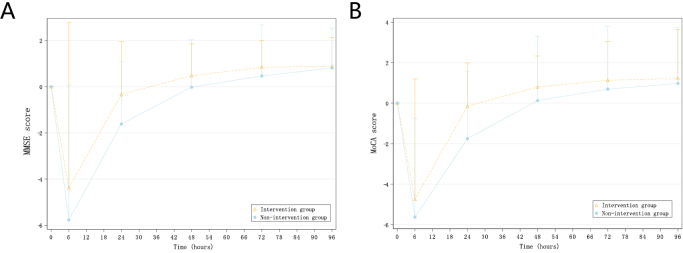
Line chart of the average MMSE/MoCA score change (from baseline) in the intervention group and the nonintervention group at each visit. Abbreviations: MMSE, mini-mental state examination; MoCA, Montreal cognitive assessment scale (A). Line chart of the average MMSE score changes (from baseline) at each visit. (B) Line chart of the average MoCA score changes (from baseline) at each visit. Error bars indicate standard deviation.

The baseline and postoperative MMSE score at every visit were presented in Table 2. The postoperative mean MMSE score in the intervention group was significantly higher than that in the non-intervention group at each visit. The mean (SD) postoperative AUC_0–96h_ of MMSE total scores were 2770.67 (150.67) for intervention group and 2651.34 (257.41) for nonintervention group (*P* = 0.010). A significant difference of AUC_0–96h_ for the change in total MMSE score from baseline was found between two groups (intervention group: −2.67 [170.29]; non-intervention group: −53.05 [164.04], *P* = 0.021) (Figure 2A), while the sensitivity analysis corroborates the main analysis conclusions (Supplemental Digital Content eTable 1, available at: http://links.lww.com/JS9/G247). In the AUC_0–96h_ comparison of MMSE sub-score, differences showed in attention and calculation (intervention group: 451.00 [69.09]; nonintervention group: 412.92 [102.50], *P* = 0.024), verbal expressions (intervention group: 90.00 [17.48]; nonintervention group: 72.59 [34.92], *P* = 0.012), and graphic drawing (intervention group: 91.58 [11.53]; nonintervention group: 81.07 [23.34], *P* = 0.015) (Supplemental Digital Content eTable 2, available at: http://links.lww.com/JS9/G248). However, AUC_0–96h_ of MMSE sub-score change from baseline showed no difference in any item.

**Table 2:** Summary of primary and secondary outcomes between the intervention group and non-intervention group.

Score	Mean (SD)		Least-squares mean^a^ (95% CI)	*P-*value
	Intervention group (*N* = 58)	Nonintervention group (*N* = 65)		
Primary outcome				
MMSE score				
Baseline	28.5 (2.1)	28.0 (2.7)	NA	0.608
Postop 6 h	24.1 (7.0)	22.5 (6.4)	1.5 (−1.4 to 4.3)	0.042
Postop 24 h	28.2 (2.9)	26.4 (3.8)	1.3 (0.3 to 2.4)	0.011
Postop 48 h	29.3 (1.4)	28.1 (2.7)	0.8 (0.1 to 1.4)	0.021
Postop 72 h	29.7 (1.0)	28.7 (2.4)	0.7 (0.01 to 1.3)	0.006
Postop 96 h	29.8 (1.0)	29.0 (2.3)	0.4 (−0.2 to 0.9)	0.026
AUC_0–96h_, from baseline	−2.7 (170.3)	−53.1 (164.0)	72.1 (1.6 to 142.5)	0.021
Secondary outcomes				
MoCA score				
Baseline	24.1 (3.1)	23.5 (4.2)	NA	0.813
Postop 6 h	19.4 (6.6)	18.2 (5.8)	0.9 (−1.5 to 3.3)	0.120
Postop 24 h	24.0 (3.7)	21.8 (4.9)	1.7 (0.5 to 2.9)	0.057
Postop 48 h	25.3 (2.2)	23.8 (3.8)	1.0 (0 to 1.9)	0.185
Postop 72 h	25.8 (1.3)	24.5 (3.5)	0.9 (−0.1 to 1.8)	0.065
Postop 96 h	25.8 (0.9)	24.7 (3.1)	0.8 (−0.01 to 1.6)	0.022
AUC_0-96h_, from baseline	10.5 (156.0)	−26.3 (219.2)	67.5 (−4.5 to 139.4)	0.190
PNCD, No. (%)^b^	18 (31.0)	36 (55.4)	NA	0.005
Postop hospital stays, day	4.9 (1.5)	4.9 (1.3)	NA	0.898

^a^ The least squares means with 95% confidence interval is estimated using analysis of covariance, with baseline as a covariate and group as a fixed effect.

^b^ In the intervention group and non-intervention group, 16 and 15 patients, respectively, could not be determined for PNCD due to missing postoperative MMSE assessments, but were still included in the total number, according to the intention-to-treat (ITT) principle.

The subgroup analysis associated with gender, age, BMI and surgical procedure were reported in Supplemental Digital Content eTable 3, available at: http://links.lww.com/JS9/G250. In patients older than 65 years, the AUC_0-96h_ of the change from baseline in total MMSE scores exhibit statistically significant differences between groups (intervention group: 42.0 [242.8]; non–intervention group: −101.4 [194.5], *P* = 0.038, *P* for interaction = 0.031).

### Secondary outcomes

The mean (SD) of the AUC_0-96h_ for the total MoCA scores were 2373.17 (175.07) for the intervention group and 2244.80 (336.78) for the non-intervention group, with no statistically significant difference between groups (P = 0.2110). The AUC_0-96h_ for the change from baseline in total MoCA scores were 10.5 (156.0) for the intervention group and −26.3 (219.2) for the non-intervention group, also demonstrating no statistically significant difference (*P* = 0.190). Nevertheless, changes from baseline in MoCA scores at each visit in the intervention group and the nonintervention group showed a similar tendency of the result of MMSE (Figure 2B). We assessed the incidence of PNCD using the MMSE total score, and observed significant differences between intervention group (31.0%) and non-intervention group (55.4%) (*P* = 0.005), which represents an Absolute Risk Reduction (ARR) of 24.4% and a Number Needed to Treat (NNT) of 4.1. Subgroups analysis of PNCD found no interaction between characteristics and drug effect (Supplemental Digital Content eFigure 1, available at: http://links.lww.com/JS9/H44). The post-hoc power analysis of main results is shown in Supplemental Digital Content Table 4, available at: http://links.lww.com/JS9/G251.

A total of 277 treatment-emergent adverse events (TEAEs) observed in 93 (93/123, 75.6%) subjects during the trial. The TEAE incidence was comparable between the intervention group (44/59, 74.6%) and the nonintervention group (49/64, 76.6%) (*P* = 0.798). All TEAEs were mild in severity. Most events resolved (intervention: 126/134 events; non-intervention: 136/143 events), while 8 (intervention) and 7 (non-intervention) events had unknown outcomes, all deemed unrelated to the study drug (Supplemental Digital Content eTable 5, available at: http://links.lww.com/JS9/G252). Two patients (3.4%) in the intervention group withdrew due to TEAEs (one case of vomiting and nausea, one case of epilepsy). No adverse drug reactions, serious TEAEs, or deaths were reported.

### Exploratory clinical outcomes

**Figure 3.**
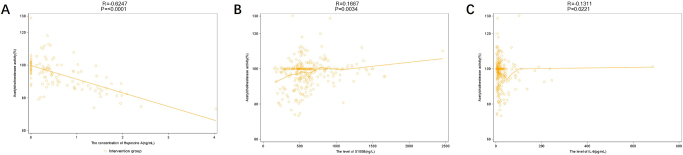
Scatter plot and Spearman’s rank correlation analysis of exploratory indicators. (A) Huperzine A and acetylcholinesterase activity in intervention group (*r* = − 0.625, *P* < 0.001). (B) Level of S100B and acetylcholinesterase activity (*r* = 0.167, *P* = 0.003). (C) IL-6 levels and acetylcholinesterase activity (*r* = − 0.131, *P* = 0.022).

The changes in exploratory indicators after injection are shown in Supplemental Digital Content eFigure 2, available at: http://links.lww.com/JS9/H44. Plasma huperzine A levels peaked at 15 minutes postfirst dose with the mean plasma concentration (SD) of 1.13 (0.72) ng/mL, followed by a gradual decline starting at 4 hours. Subsequent doses consistently induced rapid rises in plasma levels, with peak concentrations (1.24 [0.26] ng/mL at 2nd dose, 1.29 [0.93] ng/mL at 3rd dose, and 1.28 [0.83] ng/mL at 4th dose) observed within 5 minutes after each administration. Statistical analysis revealed a significant inverse correlation between serum huperzine A concentration and acetylcholinesterase (AChE) activity (Spearman’s r = − 0.625, *P* < 0.001), as illustrated in Figure 3A. However, no significant associations were detected between circulating huperzine A levels and the concentrations of neural biomarkers (neuron-specific enolase [NSE] and S100 calcium-binding protein B [S100B]) or inflammatory cytokines (interleukin-6 [IL-6] and tumor necrosis factor-alpha [TNF-α]) (*P* > 0.05 for all comparisons).

Further examination of biomarker relationships with AChE activity demonstrated differential associations. While serum NSE and TNF-α levels showed no statistically significant correlation with AChE activity, S100B concentrations demonstrated a positive correlation with AChE activity (r = 0.167, *P* = 0.003) as depicted in Figure 3B. Conversely, IL-6 levels exhibited an inverse correlation with AChE activity (r = − 0.131, *P* = 0.022), as presented in Figure 3C.

## Discussion

This trial is the first to investigate the effect of huperzine A injection on postoperative cognitive function after general anesthesia in humans. In this randomized clinical trial, the AUC_0-96h_ of postoperative MMSE total score and the AUC_0–96h_ of postoperative MMSE change from baseline in intervention group were both statistical significantly higher than nonintervention groups, indicating that postoperative intramuscular administration of huperzine A attenuated early cognitive decline in neurosurgical patients under general anesthesia. Additionally, huperzine A reduced the incidence of PNCD by 24.4%, which represents a NNT of 4.1, indicating a substantial therapeutic benefit. However, secondary outcome of MoCA scores trended similarly but did not reach statistical significance. Primarily, the discrepancy may be attributed to higher sensitivity of MoCA in mild cognitive impairment and higher rate of operational missingness in MoCA score assessment compared to MMSE in the trial[26]. Secondly, the MMSE emphasizes memory, attention and calculation, domains where Huperzine A’s cholinergic mechanism mainly affect, while the MoCA prioritizes executive function and complex abstraction, which may require longer intervention periods for detectable improvement[27]. Additionally, our analysis indicated that missing MoCA assessments were not complete random but were significantly associated with poorer cognitive performance at the prior visit (a Missing at Random mechanism). This, combined with the instrument’s greater operational complexity and the study’s reduced statistical power following early termination, likely compromised the MoCA’s ability to robustly detect a between-group difference. Thus, the MoCA results should be viewed as inconclusive rather than as evidence of no effect.

The sub-score of attention and calculation, verbal expressions, and graphic drawing showed statistically difference in AUC_0-96h_ of postoperative MMSE total score, but no difference of the MMSE change from the baseline. These sub-scores improvement may be related to acetylcholine’s role in executive function and visuospatial processing. Subgroup analysis showed that the age is the primary factor with significant interaction effect: huperzine A injection significantly reduced postoperative cognitive decline in people aged 65 years and older. However, logistic regression analysis found no evident factor with interaction between two groups. Although there are variations in results due to different analytical approaches, it is acknowledged that elderly patients exhibit higher susceptibility to PNCD during general anesthesia[28]. The therapeutic potential of huperzine A in elderly populations warrants further investigation as a key research direction.

The incidence of adverse events was similar between the intervention group and non-intervention group (74.6% vs 76.6%), indicating no significant safety concerns associated with Huperzine A administration. Crucially, all reported adverse events were mild in severity, with nausea (55.9% vs 56.3%), headache (44.1% vs 43.8%), and vomiting (35.6% vs 26.6%) representing the most common treatment-emergent symptoms across both cohorts. Notably, no severe adverse reactions, dose-limiting toxicities, or treatment-related deaths occurred during the study period. This safety profile aligns with Huperzine A’s established pharmacological characteristics as a selective acetylcholinesterase inhibitor, where cholinergic side effects are typically transient and self-limiting.

The cognitive-enhancing properties of Huperzine A have been well-documented in preclinical studies, though its clinical efficacy in cognitive impairment disorders such as Alzheimer’s disease (AD) has been inconsistent. As early as 2002, a prospective, double-blind, randomized controlled trial conducted in China found that Huperzine A could improve cognition, behavior, and mood in patients of Alzheimer’s disease[29]. In the study, the subject dosage was gradually increased from 100 μg BID to 200 μg BID, resulting in an average improvement of approximately 2.7 points on the MMSE after 12 weeks of treatment. In another double blind study, huperzine A was found effective in reducing cognitive and task switching deficits in Alzheimer’s disease patients with a 8-week treatment period with 200 μg BID[30]. However, in a subsequent Phase II trial in mild-to-moderate AD showed no cognitive benefit with 200 μg BID in the primary analysis, though some improvement was observed at 11 weeks in the 400 μg BID group in secondary analyses[31]. These discrepancies may be attributable to differences in dosage, treatment duration, and baseline disease severity. Beyond Alzheimer’s disease, the effects of Huperzine A have also varied across other cognitive disorders. For instance, a Phase II trial in patients with cognitive impairment following moderate-to-severe traumatic brain injury found no significant improvement in memory performance compared to placebo[32]. By contrast, a meta-analysis indicated that Huperzine A can improve cognitive function as an adjunctive treatment in schizophrenia[33].

Notably, our study extends these findings to a neurosurgical cohort, a population at high risk for PNCD due to prolonged anesthesia and surgical stress. The intriguing inconsistency of Huperzine A’s efficacy in prior AD trials, in contrast to our positive findings in PNCD, may be explained by fundamental differences in pathophysiology. AD involves a chronic, progressive neurodegenerative process characterized by extensive neuronal loss and the accumulation of amyloid-β and tau pathology. In this context, enhancing cholinergic function alone may be insufficient to reverse established damage. In contrast, PNCD often represents a more acute, transient disruption of neuronal networks, frequently triggered by neuroinflammation and oxidative stress in otherwise viable neurons. Here, the timely restoration of cholinergic tone, coupled with Huperzine A’s ancillary neuroprotective properties, may be sufficient to produce a significant clinical benefit. This distinction underscores that PNCD, as an acute brain dysfunction, may be a more responsive target for Huperzine A’s mechanism of action.

The cognitive benefits observed in this trial are mechanistically supported by both our pharmacological data and the established neurobiology of Huperzine A. Our exploratory analysis confirmed a significant inverse correlation between serum Huperzine A concentration and AChE activity (*r* = −0.625, *P* < 0.001), providing direct evidence of successful reversible AChE inhibition in our cohort. Increased ACh acts as a catalyst for cognitive improvement by initiating a synergistic suite of mechanisms. These include bolstering neurotrophic factor expression and synaptic activity, alongside providing neuroprotection via *N*-methyl-D-aspartate receptor antagonism and reactive oxygen species modulation, thereby ensuring neuronal survival and enhanced cognitive function^[15,16,20,34–36]^. However, the therapeutic potential of Huperzine A is not confined to cholinergic enhancement. A growing body of preclinical evidence underscores its potent neuroprotective properties. Firstly, Huperzine A exhibits significant anti-inflammatory effects by suppressing the transcriptional activation of the NF-κB signaling pathway, thereby decreasing the expression of pro-inflammatory cytokines such as IL-1β and TNF-α^[37–39]^. Secondly, Huperzine A has demonstrated anti-apoptotic and antioxidant capabilities. It can upregulate anti-apoptotic proteins like Bcl-2 and GPX4, and downregulate pro-apoptotic factors such as Bax and p53, thereby protecting neurons from programmed cell death induced by various insults^[40,41]^.To further investigate the pathophysiology of PNCD and potential mechanisms beyond direct cholinergic enhancement, we measured a panel of exploratory biomarkers. NSE and S100B were selected as markers of neuronal injury, while IL-6 and TNF-α were chosen as indicators of the systemic inflammatory response, all based on prior evidence linking their perioperative elevations to PNCD. In our study, the postoperative rise in NSE, S100B, and IL-6 was attenuated in the Huperzine A group, suggesting a potential biological effect. However, it is critical to note that exploratory analyses revealed no significant correlations between serum Huperzine A concentration and these biomarkers. The lack of correlation may be attributable to the limited sample size or the predominant role of the cholinergic pathway in the acute postoperative setting. Further animal and human studies are needed to elaborate the mechanism of huperzine A *in vivo*.

## Limitations

This study has several limitations that should be considered when interpreting the results. First, the trial was terminated prematurely due to external circumstances, resulting in a smaller-than-planned sample size. Although the primary outcome remained statistically significant, the reduced sample size limited the statistical power for several secondary and exploratory analyses, particularly those involving change-from-baseline scores and biomarker correlations. Second, the open-label design, while logistically necessary in this setting, introduces the possibility of performance and detection bias. Although cognitive assessments and laboratory analyses were performed by blinded personnel, the lack of patient and clinician blinding remains a methodological constraint. Third, the study protocol stipulated the discontinuation of cognitive testing once a ceiling MMSE score of 30 was reached, with subsequent missing MoCA scores imputed as 26. While this approach was intended to reduce patient burden, it may have introduced a conservative bias and diminished the sensitivity of the MoCA instrument to detect between-group differences. Fourth, missing data, partly exacerbated by COVID-19-related disruptions, was not entirely random. Post hoc analysis suggested that missingness at several timepoints was related to prior cognitive performance, indicating a potential for bias under a missing-at-random mechanism. Finally, as a single-center trial conducted in an East Asian population, the generalizability of the findings to other healthcare settings or ethnic groups requires further validation. Despite these limitations, the study provides promising preliminary evidence supporting the potential of Huperzine A in mitigating early postoperative cognitive decline. Future research should include multicenter, double-blind, placebo-controlled trials with extended follow-up (≥30 days) to confirm efficacy, evaluate long-term outcomes, and establish optimal dosing strategies.

## Conclusions

In this randomized trial, postoperative Huperzine A was associated with mitigated early cognitive decline and improved MMSE scores after neurosurgery. These preliminary findings suggest that Huperzine A may be a promising intervention for PNCD, but large-scale, multicenter, randomized, double-blind, placebo-controlled trials are required to confirm these findings and to evaluate the long-term benefits and optimal dosing regimen of Huperzine A in preventing PNCD.

## Data Availability

Not applicable.

## Research registration unique identifying number (UIN)

The trail was registered before patient enrolment at Chinese Clinical Trial Registry (Registration number: ChiCTR2000034272).
